# Minimally Invasive Approach to Intrauterine Device Migration Into the Bladder Using Holmium Laser

**DOI:** 10.7759/cureus.87377

**Published:** 2025-07-06

**Authors:** Luis Enrique Torres Zapata, Jose Luis Maldonado Calderón, Luis Fernando Aguilar Urrea, Aldo Missael Garcia Bailón, Víctor Manuel Molgado Garza

**Affiliations:** 1 Urology, Hospital Universitario Dr. José Eleuterio González, Universidad Autónoma de Nuevo León, Monterrey, MEX

**Keywords:** copper intrauterine device, endoscopic removal, holmium:yag laser, intravesical foreign body, migrated intrauterine device, minimally invasive surgery, recurrent urinary tract infection, urinary bladder

## Abstract

The intrauterine device (IUD) is a widely utilized method of contraception known for its high efficacy, safety profile, and long-term effectiveness. Despite its favorable characteristics, rare but potentially serious complications may occur, such as uterine perforation and device migration into adjacent pelvic or abdominal structures. One of the less frequent but clinically relevant complications is intravesical migration, where the device perforates the uterine wall and erodes into the urinary bladder. This can result in chronic lower urinary tract symptoms, recurrent urinary tract infections, hematuria, pelvic discomfort, and, in some cases, stone formation around the foreign body. We present the case of a 26-year-old female patient who developed recurrent urinary symptoms and intermittent hematuria three years after IUD placement. The device had not been visualized during gynecological follow-up and remained undetected during two full-term pregnancies. A noncontrast abdominal CT scan ultimately revealed a calcified IUD located within the urinary bladder. The patient underwent successful transurethral endoscopic removal using holmium:YAG laser lithotripsy in dusting mode to fragment the calcifications, followed by retrieval of the intact device with foreign body forceps. The procedure was completed without complications, and the patient reported full resolution of symptoms during follow-up. This case underscores the importance of considering IUD migration as a differential diagnosis in women presenting with unexplained urinary symptoms and a remote history of IUD use. It also demonstrates that holmium laser-assisted endoscopic management provides a safe, effective, and minimally invasive approach to remove encrusted or calcified intravesical IUDs, avoiding the need for open or laparoscopic surgery.

## Introduction

The intrauterine device (IUD) is one of the most widely used contraceptive methods worldwide, particularly the T-shaped copper IUD, due to its high efficacy, low cost, and long duration of action [1-5]. It is estimated that approximately 14.3% of women of reproductive age use this method [6]. Although it has a favorable safety profile, it is not without complications. The most common include abnormal uterine bleeding, dysmenorrhea, ectopic pregnancy, infections, and, in rare cases, uterine perforation and migration to adjacent organs [7]. The IUD is normally positioned within the uterine cavity, separated from the bladder by the uterine wall. Migration occurs when the device perforates through the uterine wall (myometrium) and erodes into adjacent organs. Also, uterine perforation can occur during insertion or as a result of progressive erosion through the myometrium. Among the main risks of device migration and perforation is the lack of experience on the part of healthcare personnel. This complication has an estimated incidence of 1 to 2 per 1000 insertions [8]. The most frequently reported migration sites include the pouch of Douglas, omentum, mesentery, colon, and, in rare instances, the urinary bladder [9]. Intravesical migration is particularly uncommon, with only a limited number of cases described in the medical literature. The bladder's proximity to the uterus makes it a potential site for device migration [4]. Clinically, patients may present with nonspecific urinary symptoms such as dysuria, hematuria, urinary frequency, or recurrent urinary tract infections, which may delay diagnosis [3,9]. In such cases, ultrasound may be insufficient, and computed tomography (CT) is considered the most accurate tool for localizing the foreign body and evaluating its relationship with surrounding bladder structures [10]. In most reported cases, the IUD is partially or completely calcified due to prolonged exposure to urine [11]. The treatment of choice is device removal. Surgical approaches have evolved from open cystolithotomy to minimally invasive techniques such as endoscopic holmium:YAG laser-assisted excision. Particularly in cases with significant calcifications, endoscopic laser excision offers benefits such as outpatient management, immediate recovery without wounds or scarring, minimal bleeding, and reduced surgical time. These differences highlight the advantage of a minimally invasive approach using holmium laser energy.

This article presents a rare case of IUD migration into the urinary bladder with a prolonged clinical course, which was successfully treated with endoscopic holmium laser removal. Minimally invasive alternatives for device removal are worth considering, as they reduce complications and achieve better outcomes. Endourological removal is a highly effective method for minimally invasive device removal. The objective of this case report is to raise awareness about the possibility of IUD migration into the bladder and demonstrate successful endourological management.

## Case presentation

A 26-year-old female patient with no family history of chronic degenerative diseases was evaluated. She denied smoking, alcohol consumption, substance abuse, and allergies. Her medical history was notable for recurrent urinary tract infections and intermittent nonclotting hematuria of three years’ duration, with spontaneous remissions and relapses. Gynecological-obstetric history included gravida 2, para 2, and abortion 1 (G2P2A1). She had an IUD placed in 2020 at a health center.

Her symptoms began one week after IUD insertion with dysuria, urinary frequency, urinary urgency, and nonclot-forming hematuria, which remitted and recurred spontaneously over the course of six months, which is why she decided to seek medical attention. She consulted a gynecologist, where the IUD was not visualized. At that time, a 16-week pregnancy was diagnosed. During pregnancy, she continued experiencing hematuria, which was initially misattributed to threatened abortion. She also suffered recurrent urinary tract infections treated with beta-lactam antibiotics. The pregnancy progressed to term and resulted in a eutocic delivery. One year later, she had a second uncomplicated pregnancy with normal delivery, followed by an incomplete abortion in 2022.

In January 2023, her symptoms worsened, with persistent hematuria, hypogastric pain exacerbated during urination, and febrile episodes. An abdominal ultrasound performed at a private clinic showed a hyperechoic image in the bladder, leading to a referral to our institution.

During the evaluation, she was afebrile, had stable normal vital signs, and had no cardiorespiratory compromise. Her abdomen was moderately tender, with no evidence of peritoneal irritation or perforation. Her extremities were symmetrical and eutrophic, with no abnormalities. The genitals and vagina showed no signs of atrophy or visible lesions and absence of IUD on gynecologic exam.

Laboratory studies

**Figure 1:**
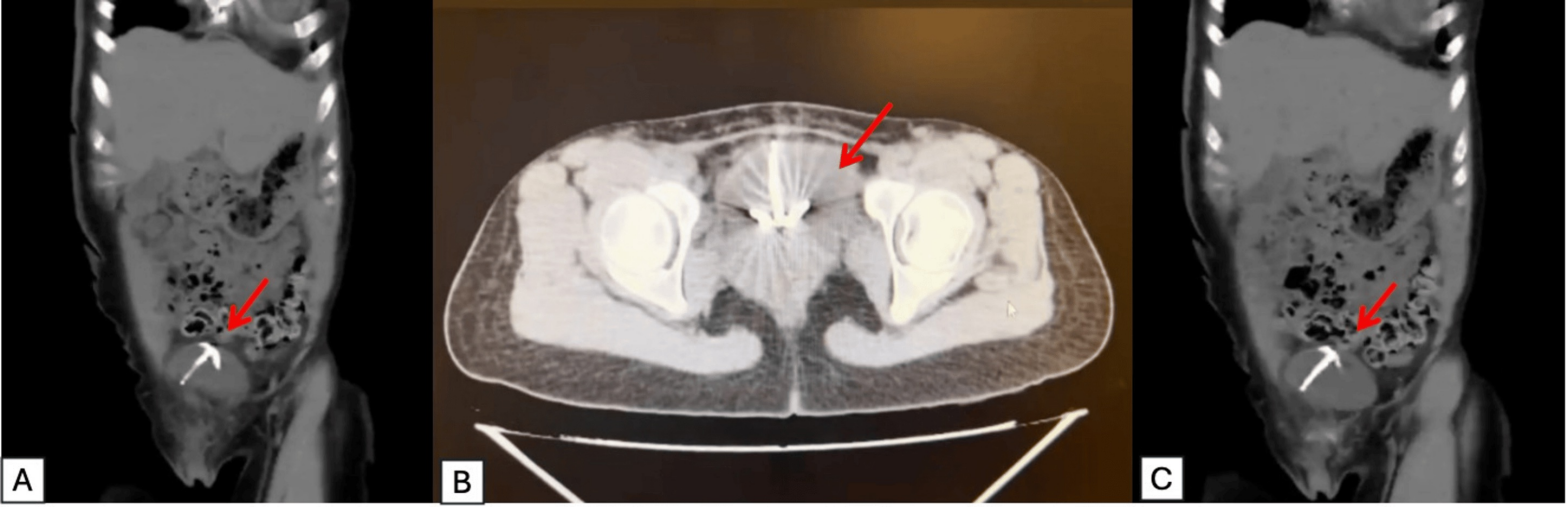
(A,C) CT noncontrast showing IUD with red arrow, coronal section. (B) Axial section CT: computed tomography; IUD: intrauterine device

Blood count included hemoglobin (HGB), 12.0; white blood cell (WBC), 7; platelet (PLT), 350; chemistry: glucose (Glu), 80. Blood urea nitrogen (BUN) was 12, and creatinine (Cr) was 0.5. Serum electrolytes were as follows: chloride (Cl), 100; sodium (Na), 140; potassium (K), 3.6. Urinalysis nitrites were negative, leukocyte esterase was negative, bacteria were absent, and urine culture was negative. Given the suspicion of bladder calculi, a noncontrast abdominal computed tomography (CT) scan was obtained, confirming the presence of an intravesical IUD (Figures 1A-1C).

**Figure 2:**
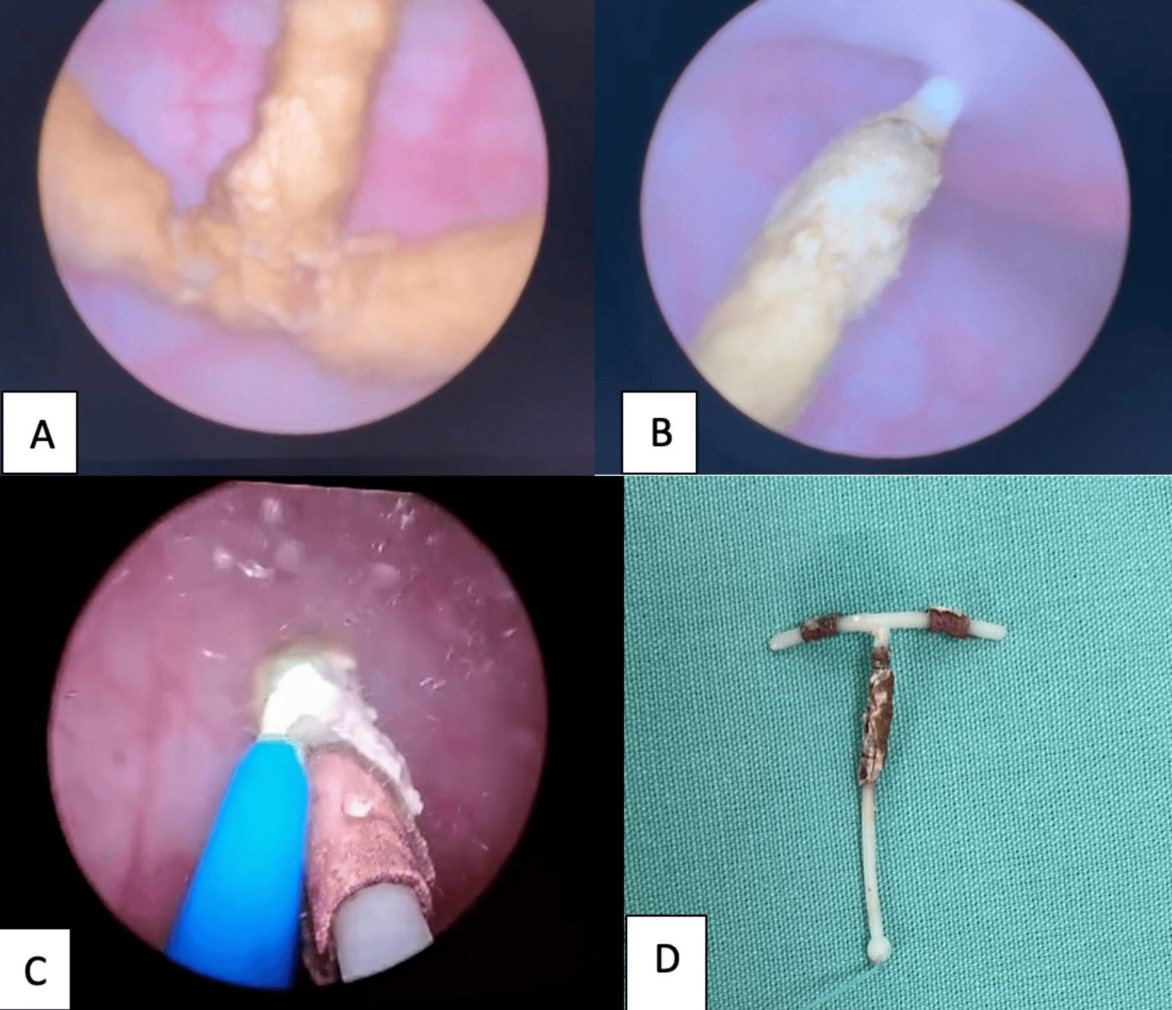
(A) Cystoscopic view of the calcified IUD (pretreatment). (B) Intrauterine device adhered to the bladder dome and holmium laser fragmentation in progress. (C) IUD release after calcification removal using holmium laser lithotripsy. (D) Removed IUD sample. Laser parameters: holmium:YAG, 0.5 J, 15 Hz, dusting mode IUD: intrauterine device

Endourological removal of the device was scheduled. Under epidural anesthesia, cystoscopy was performed using a 22 Fr sheath cystoscope. Upon entering the bladder, the mucosa appeared normal with no evidence of trabeculation. Ureteral orifices were visualized in anatomical position, with normal morphology and peristalsis. A completely calcified IUD, measuring approximately 3 cm in length and 1 cm in width, was identified and firmly adhered to the bladder dome. Fragmentation and release of the device were performed using holmium:YAG laser with the following settings: long pulses, 15 Hz, and 0.5 Joules. Laser energy was carefully applied to the areas of calcification and adhesion, allowing for progressive disintegration of the encrustation and safe release of the IUD without significant thermal injury to the mucosa. Once fully detached, the device was extracted in its entirety using grasping forceps under direct vision. The IUD was confirmed to be intact, with no residual fragments. Minimal bleeding was observed (<5 cc), and the total operative time was 90 minutes. No bladder perforation or other intraoperative complications were noted. Final inspection revealed mild edema of the bladder dome, with no evidence of transmural injury. Ureteral orifices remained unaltered. No bladder catheter was placed at the end of the procedure (Figures 2A-2D).

A ureteral stent (STU) 16 Fr was placed upon discharge and removed one week later during outpatient follow-up. At follow-up evaluation, a month later, she remained asymptomatic, with an International Prostate Symptom Score (IPSS) pain score of 0 on the visual analog scale (VAS), and was discharged. The patient was asymptomatic, with no hematuria, dysuria, hypogastric pain, or other accompanying symptoms.

## Discussion

Migration of the IUD into the urinary bladder is a rare but potentially serious complication. Although uterine perforation occurs in approximately 1 to 2 cases per 1,000 insertions, intravesical migration accounts for only a minority of these [3]. This condition is often underdiagnosed, as its symptoms are frequently nonspecific and may be misattributed to recurrent urinary tract infections or other lower urinary tract disorders [8]. In the case presented, the patient developed intermittent hematuria, dysuria, and recurrent urinary tract infections after IUD placement. The absence of the device during subsequent gynecological evaluations, combined with a subsequent pregnancy, should have raised suspicion of possible migration; that is why adequate training is important during the training of health personnel [9]. The prolonged course of urinary symptoms, extending over three years, allowed for the formation of calcified deposits on the IUD, a common finding in intravesical cases [11]. The diagnosis was confirmed via CT, which is superior to ultrasound in evaluating calcified structures and precisely locating foreign bodies within the bladder. While plain radiography may be helpful, it often provides limited information regarding mucosal involvement or the extent of encrustation. Endourological management with holmium:YAG laser enabled fragmentation of the calcified deposits using the dusting technique, which facilitated complete and minimally invasive removal of the device [12]. Although holmium laser use is well-established in the treatment of urinary lithiasis, its application in the removal of calcified intravesical foreign bodies remains sparsely reported, making this case both relevant and novel. Compared to open surgery, endoscopic approaches offer lower morbidity, shorter hospitalization, and faster recovery. Nonetheless, the choice of surgical approach depends on factors such as the degree of encrustation, the exact location of the device, and the availability of specialized equipment [13,14]. This case highlights the importance of maintaining a high index of suspicion in women with persistent urinary symptoms and a history of IUD placement, as well as the need to confirm IUD position after insertion. It also underscores the utility of the holmium laser as an effective and safe tool for the extraction of calcified foreign bodies from the bladder.

## Conclusions

Intravesical migration of an IUD is a rare complication but should be considered in women with a history of IUD placement who present with persistent urinary symptoms such as hematuria, dysuria, or recurrent urinary tract infections. Initial diagnostic evaluation should include abdominal ultrasound, complemented by CT in cases where perforation or bladder localization is suspected, particularly if calcifications are present. Endourological management using holmium:YAG laser offers a minimally invasive, safe, and effective alternative for the extraction of IUDs migrated into the bladder, especially when calcified. This technique allows the fragmentation of adherent deposits without damaging the bladder mucosa and facilitates rapid postoperative recovery. This case reinforces the importance of appropriate follow-up after IUD insertion as well as the need to confirm device location in patients where it is not visualized during subsequent gynecologic evaluations. Timely intervention prevents major urological complications and improves clinical outcomes.

## References

[1] Kaislasuo J, Suhonen S, Gissler M, Lähteenmäki P, Heikinheimo O (2013). Uterine perforation caused by intrauterine devices: clinical course and treatment. Hum Reprod.

[2] Zakin D, Stern WZ, Rosenblatt R (1981). Complete and partial uterine perforation and embedding following insertion of intrauterine devices. I. Classification, complications, mechanism, incidence, and missing string. Obstet Gynecol Surv.

[3] Markovitch O, Klein Z, Gidoni Y, Herman A, Pansky M (2002). Extrauterine mislocated IUD: is surgical removal mandatory?. Contraception.

[4] Jaziri Y, Saidani B, Saadi A, Haroun A, Chakroun M, Ben Slama R (2025). Intrauterine device migration into the bladder with urolithiasis: a case report. Int J Surg Case Rep.

[5] (2025). Family planning/contraception methods. https://www.who.int/news-room/fact-sheets/detail/family-planning-contraception.

[6] Buhling KJ, Zite NB, Lotke P, Black K (2014). Worldwide use of intrauterine contraception: a review. Contraception.

[7] Hubacher D, Lara-Ricalde R, Taylor DJ, Guerra-Infante F, Guzmán-Rodríguez R (2001). Use of copper intrauterine devices and the risk of tubal infertility among nulligravid women. N Engl J Med.

[8] Gill RS, Mok D, Hudson M, Shi X, Birch DW, Karmali S (2012). Laparoscopic removal of an intra-abdominal intrauterine device: case and systematic review. Contraception.

[9] De Silva WS, Kodithuwakku KA, Aponsu GU, Rathnayake RM, Rajasegaram E (2017). A large bladder stone caused by the intravesical migration of an intrauterine contraceptive device: a case report. J Med Case Rep.

[10] El-Hefnawy AS, El-Nahas AR, Osman Y, Bazeed MA (2008). Urinary complications of migrated intrauterine contraceptive device. Int Urogynecol J Pelvic Floor Dysfunct.

[11] Atakan RH, Kaplan M, Ertrk E (2013). Intravesical migration of an intrauterine device with stone formation. Korean J Urol.

[12] Rasyid N, Nainggolan HJ, Jonardi PA, Raharja PA, Wiweko B, Atmoko W, Birowo P (2021). Early-onset complete spontaneous migration of contraceptive intrauterine device to the bladder in a post C-section patient: a case report. Int J Surg Case Rep.

[13] Houmaid H, Harou K, Fakhir B (2024). Migration of an intrauterine contraceptive device into the bladder complicated by stone formation an exceptional complication: case report and literature review. Contracept Reprod Med.

[14] Benaguida H, Kiram H, Telmoudi EC, Ouafidi B, Benhessou M, Ennachit M, Elkarroumi M (2021). Intraperitoneal migration of an intrauterine device (IUD): a case report. Ann Med Surg (Lond).

